# Plasma Folate and Vitamin B_12_ Levels in Patients with Hepatocellular Carcinoma

**DOI:** 10.3390/ijms17071032

**Published:** 2016-06-30

**Authors:** Lian-Hua Cui, Zhen-Yu Quan, Jin-Mei Piao, Ting-Ting Zhang, Meng-Hui Jiang, Min-Ho Shin, Jin-Su Choi

**Affiliations:** 1 Department of Public Health, Qingdao University Medical College, No. 38 Dengzhou Road, Qingdao 266021, China; jinmeipark@163.com (J.-M.P.); guti2016@163.com (T.-T.Z.); menghuidream@163.com (M.-H.J.); 2 Department of Public Health, Yanbian University Medical College, No. 977 Gongyuan Road, Yanji 133002, China; zyquan@ybu.edu.cn; 3 Department of Oncology, Shengli Oil-Field Central Hospital, No. 31 Jinan Road, Dongying 257000, China; 4 Department of Preventive Medicine, Chonnam National University Medical School, Gwangju 501-746, Korea; mhshinx@paran.com (M.-H.S.); jschoix@jnu.ac.kr (J.-S.C.)

**Keywords:** folate, vitamin B_12_, hepatocellular carcinoma, susceptibility, tumor progression

## Abstract

Folate and vitamin B_12_ involved in the one-carbon metabolism may play a key role in carcinogenesis and progression of hepatocellular carcinoma (HCC) through influencing DNA integrity. The purpose of this study is to evaluate the association of plasma folate and vitamin B12 levels with HCC in a case-control study on 312 HCC patients and 325 cancer-free controls. Plasma concentrations of folate and vitamin B_12_ in all the subjects were measured by electrochemiluminescence immunoassay. Meanwhile, the information of HCC patients’ clinical characteristics including tumor-node-metastasis (TNM) stage, tumor size and tumor markers were collected. The patients of HCC had significantly lower folate levels than those of controls; there was no significant difference in the mean of plasma vitamin B_12_ levels. We also observed an inverse association between the levels of plasma folate and HCC: the adjusted odds ratios (OR) (95% confidence intervals (CI)) of HCC from the highest to lowest quartile of folate were 0.30 (0.15–0.60), 0.33 (0.17–0.65), and 0.19 (0.09–0.38). Compared to the subjects in the lowest quartile of plasma vitamin B_12_, only the subjects in the highest quartile of vitamin B_12_ exhibited a significant positive relationship with HCC, the adjusted OR was 2.01 (95% CI, 1.02–3.98). HCC patients with Stage III and IV or bigger tumor size had lower folate and higher vitamin B_12_ levels. There was no significant difference in the mean plasma folate levels of the HCC cases in tumor markers status (AFP, CEA and CA19-9 levels), whereas patients with higher CEA or CA19-9 levels retained significantly more plasma vitamin B_12_ than those with normal-CEA or CA19-9 level. In conclusion, plasma folate and vitamin B_12_ levels could be associated with HCC, and might be used as predictors of clinical characteristics of HCC patients. However, further prospective studies are essential to confirm the observed results.

## 1. Introduction

Primary liver cancer is the fifth most common cancer in the world, and the third common cause of cancer mortality. According to previous scientific research, chronic hepatitis B viral infection is the primal cause of hepatocellular carcinoma (HCC) in Asia and Africa. Recently, growing evidence indicates that there may be an relationship between the factors of environment or nutrition with the development of HCC [1,2]. The levels of plasma folate and vitamin B_12_ maybe one of the important factors. Folate and vitamin B_12_ as water-soluble B vitamins, which are involved in the one-carbon metabolism are essential for DNA methylation, synthesis and repair during cell regeneration. Therefore, recently, the function of folate and vitamin B_12_ in carcinogenesis has attracted more attention from researchers [3,4,5].

The folate, as a natural anti-cancer vitamin, is found to induce the apotosis of cancer cells and affect the gene expression of cancer cells [6,7]. Vitamin B_12_ involved in one-carbon metabolism is dependent on methylation of homocysteine to form methionine by methionine synthase. Therefore, we propose that vitamin B_12_ contributes to an alteration of plasma homocysteine level and folate status, which may be important in tumorigenesis. 

Liver is the major site of storage and metabolism for folate and vitamin B_12__,_ which are involved in the methylation and synthesis of macromolecules. Recent animal experiment showed a significant reduction in global DNA methylation with folate deficient diet, which promotes the initiation of carcinogenesis. Given the importance of folate in DNA methylation and synthesis, chronic folate deficiency may induce genome-wide DNA hypomethylation [8,9]. In addition, a recent study reported that approximately 60% of HCC patients were deficient in folate and folate levels decreased drastically as HCC progressed [10]. Previous studies also proved that folate depletion and elevated plasma vitamin B_12_ for other reasons, such as oxidative stress, hepatitis and alcoholic liver disease [11,12,13], may lead to neoplasia and HCC development. 

Based on the above, we hypothesized that human plasma folate and vitamin B_12_ levels might be related to HCC development. Therefore, a case-control study (including 312 HCC cases and 325 controls) was designed to evaluate the effect of plasma folate and vitamin B_12_ on the development and clinical pathological parameters of HCC, including tumor stage, tumor size and tumor markers.

## 2. Results

### 2.1. Demographic Analyses of Serum Folate and Vitamin B_12_ Levels

The baseline characteristics of the studied population were summarized in Table 1. A total of 312 cases and 325 controls were included in these analyses. The HCC patients and control subjects appeared to be adequately matched on age, sex, smoking and drinking, but these data showed no significant differences (*p* > 0.05). However, the percentage of subjects carrying hepatitis B surface antigen (HBsAg) was obviously higher in these cases (84%) when compared to the controls (6.2%), and this difference was statistically significant (*p* = 0.000). Furthermore, a statistically significant difference on plasma folate was also found in the case group, with a lower mean plasma folate level and more folate deficient subjects. However, although the proportion of vitamin B_12_ deficient subjects in HCC cases was higher compared to the healthy controls (*p* = 0.016), there was no significant difference in geometric mean of plasma vitamin B_12_ levels (*p* = 0.397).

### 2.2. Plasma Folate and Vitamin B_12_ Levels in Relation to HCC Risk

To analyze the relationship between plasma folate and vitamin B_12_ levels with HCC, plasma folate and vitamin B_12_ levels were categorized into quartiles based on the distribution in the controls. The quartile cut points for each nutrient were: folate: 2.2–8.8, 8.9–12.2, 12.3–15.8, 15.8–45.4 (nmol/L); vitamin B_12_: 227–265, 266–406, 407–589, 590–1478 (pmol/L). After being adjusted by age, sex, smoking and drinking, HBsAg, plasma folate level displayed an inverse association with HCC in the basic conditional logistic regression models (Table 2), this was most evident when comparing the highest versus lowest quartile (adjusted OR = 0.19; 95% CI = 0.09–0.38). Compared to the subjects in the lowest quartile of plasma vitamin B_12_, the subjects in the highest quartile exhibited a significant positive association of HCC (adjusted OR = 2.01, 95% CI = 1.02–3.98). No significant interaction effect was observed between plasma folate and vitamin B_12_ on HCC.

### 2.3. Tumor Stage, Tumor Size and Tumor Markers in Hepatocellular Carcinoma Patients with Various Folate and Vitamin B12 Levels

As shown in Table 3, we analyzed the mean of plasma folate and vitamin B_12_ status in the classified HCC patients, which are sorted based on clinical features including tumor stage, tumor size and tumor markers (AFP, CEA and CA19-9) after being adjusted for age, sex, smoking and drinking, HBsAg. When HCC patients were categorized into stages I + II and III + IV, HCC patients with Stage III + IV had lower levels of folate and higher plasma vitamin B_12_ levels than patients with stages I + II. Similarly, plasma folate and vitamin B_12_ levels exhibited the same relation with tumor size of HCC. Compared to patients with tumor size smaller than 5 cm, patients with tumor size bigger than or equal to 5 cm displayed significantly lower plasma folate level (9.5 versus 10.5 nmol/L; *p* = 0.047) and tremendously higher plasma vitamin B_12_ level (419.9 versus 347.2 pmol/L; *p* = 0.007). There was no significant difference in the mean plasma folate level of the HCC cases in tumor markers status (AFP, CEA and CA19-9 levels). However, patients with a higher CEA or CA19-9 level retained significantly more plasma vitamin B_12_ than those with a normal CEA or CA19-9 level (CEA: *p* = 0.002; CA19-9: *p* = 0.001).

### 2.4. Stratified Analyses of Odds Ratios and 95% Confidence Intervals of HCC with Plasma Folate and Vitamin B_12_

To further evaluate the association of likelihood of HCC risk with plasma folate, we performed a stratified analysis by drinking status (Table 4). In the subgroup of non-drinking, the results revealed that the risk of HCC decreased with the increase of folate levels. The ORs and 95% CIs were 0.19 (0.08–0.49), 0.21 (0.08–0.53) and 0.12 (0.04–0.32) for subjects in the second, third and highest quartile of plasma folate, respectively, as compared with those in the lowest quartile. Whereas, in the subgroup of drinking, only the highest quartile of plasma folate exhibited a significant association with a reduction in HCC risk, with the adjusted ORs and 95% CIs being 0.24 (0.09–0.66).

Table 5 shows the results of stratified analyses by drinking status of HCC with plasma vitamin B_12_. For the non-drinking subgroup, plasma vitamin B_12_ was not significantly associated with the risk of HCC. Whereas, plasma vitamin B_12_ levels were positively associated with HCC risk in the drinking subgroup (multivariable OR = 5.60, 95% CI = 1.81 to 17.39 for highest versus lowest quartile).

## 3. Discussion

Folate, as a methyl donor in the synthesis of methionine, has been shown to mediate carcinogenesis by participating in DNA synthesis, repair, and methylation. The methionine cycle that provides one-carbon moiety for cellular methylation reactions is also dependent on vitamin B_12_. However, the epidemiological evidence regarding associations of plasma folate and vitamin B_12_ with HCC risk have not been well studied. In this current case-control (312 cases and 325 controls) study, we observed an inverse association between the levels of plasma folate and HCC, whereas only the subjects in the highest plasma vitamin B_12_ quartile exhibited a significant positive relationship with HCC. The levels of plasma folate and vitamin B_12_ were related with clinical characteristics of HCC patients.

The reduction of plasma folate in HCC patients may have resulted from various factors. For example, the most common factors are under-nutrition and severe catabolic status which cancer patients suffer from, compromising the plasma folate status [14]. Moreover, a low folate status was reported to lead to liver damage through oxidative stress and pro-fibrogenic effect [15,16]. As a major source of dietary methyl groups, folate is involved in various biological processes, including methylation, DNA synthesis, and DNA repair. Therefore, folate deficiency aids the incorporation of uracil into the DNA, which can lead to DNA breaks and chromosome instability; such breaks could contribute to the increased risk of cancer. In addition, inadequate folate may result in global DNA hypomethylation and aberrant hypermethylation in gene promoters, which subsequently interfere with gene expression and DNA repair, finally leading to tumorigenesis [17].

Our findings are strongly supported by previous studies on experimental animals, which reported an increased risk for hepatocarcinogenesis in folate-deficient mice [11,12,13]. Circulating concentrations of folate have been investigated in relation to multiple cancers, but few studies have been conducted to evaluate the relationship between plasma folate and HCC patients. In agreement with our results, Welzel et al. [18] researched a prospective high-risk cohort in Haimen City and indicated that higher folate levels in red blood cells were associated with reduced risk of hepatocarcinogenesis. Consistently, Lin CC et al. [19] showed that plasma level of folate in HCC patients was significantly lower compared to healthy controls, in spite of very small sample size in their study (40 cases and 20 controls). Recently, Cheng et al. [20] showed that HCC patients at preresection had lower serum folate than that of control groups. Chang et al. [21] also observed an inverse association between plasma folate levels and liver cancer. In fact, in other malignancies such as lung cancer [22], breast cancer [23], esophageal squamous cell carcinoma [24], and cervical cancer [25], studies have also reported a protective association between the plasma folate levels and the risk of some cancers. In addition, in a cohort study from the U.S. population of the National Health and Nutrition Examination Survey [26], results also showed that high serum and RBC folate in older adults were inversely associated with the risk of cancer incidence. Furthermore, a meta-analysis of 83 case-control studies involving 35,758 individuals also suggested that folate deficiency associated with increased overall risk of carcinogenesis [27].

Excessive alcohol consumption is a well-established risk factor for HCC [28,29,30], while alcohol could also affect folate and DNA methylation pathways by promoting the degration, and inhibiting the absorption and metabolism of this nutrient. Similarly, vitamin B_12_ deficiency is found in chronic alcoholics. Thus, we further conducted a stratified analysis by alcohol drinking status, and the results indicated that HCC risk has an inverse association with all quartiles of plasma folate levels in the subgroup of non-drinking. In the subgroup of drinking, only the highest quartile of plasma folate exhibited a significant association with the reduced of HCC risk. Persson et al. [30] suggested that higher folate intake might ameliorate the effect of alcohol consumption on the development of HCC, they observed that the individuals who consumed more than 3 drinks per day were associated with a significantly increased risk of HCC in the lower tertile of the folate intake group, but no association between alcohol consumption and HCC in the highest tertile of the folate intake group [30].

Liver is the major storage site for vitamin B_12_. Vitamin B_12_ is an important co-factor in folate metabolism but studies relating plasma vitamin B_12_ to HCC risk and prognosis are relatively very few, and the association remains vague. In this study, although a higher percent of vitamin B_12_ deficiency was observed in the HCC patients, there was no significant difference in the mean of plasma vitamin B_12_ level between patients and healthy controls. Interestingly, compared to the subjects in the lowest quartile of plasma vitamin B_12_, the subjects in the highest quartile significantly displayed increased HCC risk only in the drinking subgroup, but not in the non-drinking subgroup. However, there are few data on the effects of plasma vitamin B_12_ on the risk of HCC. A study on HCC in Taiwan stated that plasma level of vitamin B_12_ in HCC patients was significantly lower compared to healthy controls [31]. Whereas, another study [32] reported that vitamin B_12_ and related proteins displayed elevations in both HCC and chronic liver disease patients compared to controls. These different results might be due to the small sample sizes in the studies, differences in the methods of measuring vitamin B_12_ level, differences in ethnicity between the subjects, or differences in tumor stages of HCC. Our data showed no significant interaction effect between plasma folate and vitamin B_12_ on HCC.

Apart from the revelation of abnormal folate and vitamin B_12_ levels in the blood of HCC patients, we also explored the influences of the folate and vitamin B_12_ on clinical pathological parameters of HCC. In the analysis, we found that when tumor stage of HCC was categorized into stages I + II and III + IV, the tumor stage was associated with lower plasma folate level. The tumor size also displayed a similar association with folate. A recent study reported that folate status in HCC patients decreased as HCC progressed and that low blood folate status could be a risk factor for tumor progression [10]. Consistently, our data confirmed that low folate level contributes to HCC progression in tumor stage and tumor size. However, there was no significant difference in plasma folate level in the HCC cases among different tumor markers status (AFP, CEA and CA19-9 levels).

Contrary to plasma folate analysis, elevated plasma vitamin B_12_ levels might have a better prognostic significance in HCC patients. The size of the tumor nodule, which represents tumor burden, was also frequently associated with the aggressiveness of HCC [33]. CEA and CA199 have been used as tumor markers, and they were also associated with the severity of liver disease [34]. We observed that high plasma vitamin B_12_ levels were not only associated with tumor stage and tumor size but also CEA and CA19-9 levels; these results indicated that high plasma vitamin B_12_ levels could be a risk factor for tumor progression. Lin et al. [31] showed that serum vitamin B_12_ levels were positively correlated with AFP levels and tumor size, and determinants of plasma vitamin B_12_ level in HCC patients were shown to be liver injuries, and tumor progression. In addition, previous studies showed that vitamin B12 levels above 400 pg/mL reduced micronucleus formation in peripheral blood lymphocytes [35,36] and uracil misincorporation into leukocyte DNA [37]. Furthermore, Arendt et al. [38] showed that high plasma vitamin B_12_ levels increased the risk of subsequently diagnosed cancer, and cancer patients with elevated plasma vitamin B12 levels had higher mortality than those with normal vitamin B_12_ levels [39,40].

As an important coenzyme of methionine synthase, the deficiency of vitamin B_12_ led to aberrant DNA methylation and subsequent hepato-carcinogenesis. After that, the injury to the hepatocyte accelerated with tumor progression. As the liver has a crucial impact on the storage, transport and metabolism of cobalamin, the damage to liver cells may induce the impairment of hepatic B_12_ metabolism and release of vitamin B_12_, resulting in the elevation of plasma vitamin B_12_ level [41]. This may elucidate the mechanisms regarding discrepancy of vitamin B_12_ level in different tumor stages of HCC patients.

Due to a lack of detailed data on dietary folate and vitamin B_12_ intake, the possible effect of diet on HCC could not be adequately evaluated. Furthermore, given the limitation of a case-control study, it may be difficult to establish the timeline of exposure to disease outcome in the setting of a case-control study. In other words, it is hard to define the causal relationship between plasma folate and vitamin B_12_ with HCC. Therefore, in the future, prospective cohort studies on plasma folate and vitamin B_12_ and HCC should be designed to further ascertain this relationship.

## 4. Materials and Methods

### 4.1. Subjects

The study included 312 HCC patients who were consecutively admitted in the Affiliated Hospital of Medical College Qingdao University and 325 controls recruited from the community around Qingdao for health examination during the time from 2009 to 2011. All enrolled patients were newly diagnosed and pathologically confirmed as HCC, taking no therapy and without any secondary or recurrent tumors. The average age of HCC patients including 262 males and 50 females was 56.78 ± 10.51 year. The tumor stages of the patients were classified according to the international tumor-node-metastasis (TNM) staging system [42]: stages I (*n* = 50), II (*n* = 99), III (*n* = 103), and IV (*n* = 60), respectively. In this study, 312 HCC patients were divided into two groups: I + II (*n* = 149) and III + IV (*n* = 163). According to the measurement on the size of the largest tumor by a physician specialized in hepatology and oncology through CT scan, the patients were classified into: smaller than 5 cm and bigger than or equal to 5 cm.

Control subjects were frequency-matched to cases by age (±5 years), and sex, which was matched case-to-control ratio of approximately 1:1 for men and women. In brief, a total of 325 healthy individuals without any history of cancer, major organ failure (e.g., heart, brain, lung, kidney, or liver) or active intravenous drug abuse were recruited as controls. The controls included 258 males and 67 females, with a median age of 58.45 ± 9.73 year. All case and control subjects provided their informed consent. This study protocol was proved by Institutional Review Board of Medical College Qingdao University, Shandong, China.

### 4.2. Blood Biochemical Determinations

The peripheral blood of subjects was collected on an empty stomach. Plasma folate and vitamin B_12_ were determined with a commercial automatic electrochemical immuno-analyzer (Roche E170) and electrochemiluminescence immunoassay (ECLIA) kit. Plasma alpha-fetal protein (AFP), carcino-embryonic antigen (CEA) and Cancer Antigen 19-9 (CA19-9) were measured by automatic electrochemical immuno-analyzer (Roche Cobas E601) and special kit. According to the clinical criteria, plasma folate level less than 6.8 nmol/L was considered deficient; vitamin B_12_ status less than 185 pmol/L was classified as deficient [43]. The manual offered the normal reference value of these tumor markers: AFP: 0~7.2 μg/L, CEA: 0~3.4 μg/L, CA19-9: 0~39 U/mL.

### 4.3. Statistical Analysis

Non-normally distributed dependent variables were first transformed using a logarithmic function. Chi-square test was used to test the differences in the distributions of demographic factors between cases and controls. Dummy variables of the median and quartile of folate and vitamin B_12_ level were created to calculate the ORs and 95% CIs (with the lowest quartile as the reference category) as an estimate of the relative risk. Dependence between the folate, vitamin B_12_ level and the risk and tumor clinical features of HCC was evaluated using logistic regression models and covariance analysis. All statistical analysis was carried out using SPSS version 13 for Windows. Statistical significance was defined as a *p* value of less than 0.05. 

## 5. Conclusions

In summary, our data indicated that the levels of plasma folate and vitamin B_12_ are likely to be associated with the progress of liver cancer. More studies with larger sample size are required to further confirm our results. 

## Figures and Tables

**Table 1:** Baseline characteristics of participants stratified by case-control status.

Baseline Characteristics	Cases (*n* = 312) *n*(%)	Controls (*n* = 325) *n*(%)	*p* Value
Age (years)			
≤50	86(27.6)	75(22.6)	0.149
>50	226(72.4)	250(77.4)	
Sex			
Male	262(84.0)	258(79.4)	0.135
Female	50(16.0)	67(20.6)	
Drinking			
Yes	125(40.1)	126(38.8)	0.438
No	187(59.9)	199(61.2)	
Smoking			
Yes	149(47.8)	131(40.3)	0.058
No	163(52.2)	194(59.7)	
HBsAg			
Positive	262(84.0)	16(6.2)	0.000
Negative	50(16.0)	242(93.8)	
Plasma folate(nmol/L)			
Geometric mean(95% CI)	9.97(9.39–10.59)	13.3(12.7–14.2)	0.000
Deficient (<6.8 nmol/L)	78(25)	6(1.8)	0.000
Plasma vitamin B_12_ (pmol/L)			
Geometric mean(95% CI)	399.4(368.7–437.0)	391.5(368.7–419.9)	0.397
Deficient (<185 pmol/L)	46(14.7)	28(8.6)	0.016

**Table 2:** Odds ratios (OR) and 95% confidence intervals (CI) of hepatocellular carcinoma (HCC) by quartile of plasma folate and vitamin B_12_ levels.

Interquartile Range	Cases *n*(%)	Controls *n*(%)	OR ^+^ (95% CI)	*p* Value
Plasma folate (nmol/L)				
Q1 (2.2–8.8)	119(38.1)	40(12.3)	1 (Reference)	
Q2 (8.9–12.2)	74(23.7)	85(26.2)	0.30 (0.15–0.60)	0.001
Q3 (12.3–15.8)	62(19.9)	96(29.5)	0.33 (0.17–0.65)	0.001
Q4 (15.8–45.4)	57(18.3)	104(32.0)	0.19 (0.09–0.38)	0.000
Plasma vitamin B_12_ (pmol/L)				
Q1 (227–265)	81(26.0)	77(23.7)	1 (Reference)	
Q2 (266–406)	86(27.6)	73(22.5)	1.43 (0.72–2.81)	0.306
Q3 (407–589)	59(18.9)	103(31.7)	0.63 (0.31–1.25)	0.187
Q4 (590–1478)	86(27.6)	72(22.2)	2.01 (1.02–3.98)	0.045

OR ^+^, odds ratio adjusted for age, sex, smoking and drinking, HBsAg.

**Table 3:** Folate and B_12_ status in relation to clinical pathological factors of hepatocellular carcinoma.

Clinical Characteristic	*N*	Plasma Folate (nmol/L)	*p* Value ^+^	Plasma Vitamin B_12_ (pmol/L)	*p* Value ^+^
		Geometric Mean (95% CI)		Geometric Mean (95% CI)	
TNM stage					
(1) I + II	149	10.6 (9.8–11.4)		354.2 (323.8–391.5)	
(2) III + IV	163	9.3 (8.8–9.9)	0.049	411.6 (376.2–450.3)	0.038
Tumor size					
(1) <5 cm	147	10.5 (9.7–11.2)		347.2 (317.3–383.8)	
(2) ≥5 cm	165	9.5 (8.8–10.2)	0.047	419.9 (383.8–459.4)	0.007
AFP(μg/L)					
(1) ≤7.02	81	9.7 (8.6–10.9)		354.7 (301.8–407.5)	
(2) >7.02	231	10.1 (9.4–10.7)	0.611	399.4 (361.4–432.7)	0.162
CEA(μg/L)					
(1) ≤3.4	201	10.1 (9.4–10.8)		347.2 (317.3–380.0)	
(2) >3.4	111	9.7 (8.8–10.7)	0.480	464.1 (407.5–523.2)	0.002
CA19-9 (U/mL)					
(1) ≤39	190	9.9 (9.3–10.7)		350.7 (317.3–383.8)	
(2) >39	122	9.9 (9.0–10.8)	0.876	445.9 (395.4–502.7)	0.001
Controls	325	13.3 (12.7–14.2)	0.000	391.5 (368.7–419.9)	0.397

^+^ adjusted for age, sex, smoking and drinking, HbsAg.

**Table 4:** Stratified analyses of Odds ratios and 95% confidence intervals of HCC with plasma folate (nmol/L).

Group	Plasma Folate (nmol/L)	Cases *n*(%)	Controls *n*(%)	OR ^+^ (95% CI)
No drinking	Q1 (2.2–8.8)	69(36.9)	21(10.6)	1 (Reference)
	Q2 (8.9–12.2)	49(26.2)	63(31.7)	0.19 (0.08–0.49)
	Q3 (12.3–15.8)	38(20.3)	63(31.7)	0.21 (0.08–0.53)
	Q4 (15.8–45.4)	31(16.6)	52(26.1)	0.12 (0.04–0.32)
Drinking	Q1 (2.2–8.8)	50(40.0)	19(15.1)	1 (Reference)
	Q2 (8.9–12.2)	25(20.0)	22(17.5)	0.45 (0.14–1.44)
	Q3 (12.3–15.8)	24(19.2)	34(27.0)	0.59 (0.21–1.63)
	Q4 (15.8–45.4)	26(20.8)	51(40.5)	0.24 (0.09–0.66)

OR ^+^, odds ratio adjusted for age, sex, smoking and HbsAg.

**Table 5:** Stratified analyses of Odds ratios and 95% confidence intervals of HCC with plasma vitamin B_12_ (pmol/L).

Group	Plasma Vitamin B_12_ (pmol/L)	Cases *n*(%)	Controls *n*(%)	OR ^+^ (95% CI)
No drinking	Q1 (227–265)	55(29.4)	39(19.6)	1 (Reference)
	Q2 (266–406)	46(24.6)	41(20.6)	0.97 (0.39–2.43)
	Q3 (407–589)	37(19.8)	67(33.7)	0.43 (0.17–1.07)
	Q4 (590–1478)	49(26.2)	52(26.1)	1.01 (0.41–2.48)
Drinking	Q1 (227–265)	26(20.8)	39(31.0)	1 (Reference)
	Q2 (266–406)	38(30.4)	32(25.4)	2.65 (0.90–7.76)
	Q3 (407–589)	25(20.0)	36(28.6)	1.49 (0.50–4.432)
	Q4 (590–1478)	36(28.8)	19(15.1)	5.60 (1.81–17.39)

OR ^+^, odds ratio adjusted for age, sex, smoking and HbsAg.

## Abbreviations

HCC	hepatocellular carcinoma
AFP	alpha-fetal protein
CEA	carcino-embryonic antigen
CA19-9	Cancer Antigen 19-9
TNM	tumor-node-metastasis
